# An outcome‐wide analysis of the effects of diagnostic labeling of Alzheimer's disease and related dementias on social relationships

**DOI:** 10.1002/alz.13574

**Published:** 2023-12-05

**Authors:** Takashi Amano, Cal J. Halvorsen, Seoyoun Kim, Addam Reynolds, Clara Scher, Yuane Jia

**Affiliations:** ^1^ Department of Social Work School of Arts and Sciences Rutgers University Newark Newark USA; ^2^ School of Social Work Boston College Chestnut Hill USA; ^3^ Department of Sociology Texas State University San Marcos USA; ^4^ Leonard Davis School of Gerontology University of Southern California Los Angeles USA; ^5^ School of Social Work Rutgers University New Brunswick USA; ^6^ Department of Interdisciplinary Studies School of Health Professions Rutgers Biomedical and Health Sciences Newark USA

**Keywords:** diagnostic label of dementia, Health and Retirement Study (HRS), outcome‐wide analysis, propensity score analysis, social engagement, social network, social support

## Abstract

**INTRODUCTION:**

This study examines how receiving a dementia diagnosis influences social relationships by race and ethnicity.

**METHODS:**

Using data from the Health and Retirement Study (10 waves; 7,159 observations) of adults 70 years and older predicted to have dementia using Gianattasio‐Power scores (91% accuracy), this study assessed changes in social support, engagement, and networks after a dementia diagnosis. We utilized quasi‐experimental methods to estimate treatment effects and subgroup analyses by race/ethnicity.

**RESULTS:**

A diagnostic label significantly increased the likelihood of gaining social support but reduced social engagement and one measure of social networks. With some exceptions, the results were similar by race and ethnicity.

**DISCUSSION:**

Results suggest that among older adults with assumed dementia, being diagnosed by a doctor may influence social relationships in both support‐seeking and socially withdrawn ways. This suggests that discussing services and supports at the time of diagnosis is important for healthcare professionals.

## 1 BACKGROUND

Receiving a diagnosis (“diagnostic label”) of Alzheimer's disease and related dementias (ADRD) may have a profound impact on people's lives. A diagnostic label may benefit those with ADRD and their families due to psychological relief^1^, ^2^, ^3^, ^4^, ^5^, ^6^, ^7^, ^8^, ^9^ and increased healthy behaviors,^10^ future care planning,^11^ and safety planning, such as fall prevention strategies.^12^ However, a diagnostic label may also cause psychological distress,^2^, ^13^ post‐traumatic stress,^14^ and suicidal ideation.^15^, ^16^ Given the nuanced impact of an ADRD diagnostic label, healthcare providers need to consider the best ways to support patients and their families.

The impact of a diagnostic label of ADRD on individuals’ social lives has not been well investigated. Three aspects of social relationships are often operationalized as social support, social engagement, and social networks – all theorized to be associated with health.^17^ Specifically, social support and social engagement mediate the link between social networks and health. While the provision of social support involves direct and tangible assistance extended to an individual, social engagement influences health by means of active participation in meaningful social contexts. Consequently, in comprehending the effects of a diagnostic label of ADRD on an individual's social well‐being, it becomes imperative to scrutinize these three constructs separately in the same model. Although it is well‐investigated that people with ADRD are more likely to receive social and healthcare support,^18^, ^19^, ^20^, ^21^ it is not clear to what extent a diagnostic label contributes to the increase in support. Receiving a diagnosis of ADRD may discourage people from being socially engaged through both informal and formal means.^2^, ^14^, ^22^, ^23^ However, there is a need for studies with larger sample sizes that focus on both formal and informal social activities to better estimate effects.

Despite the scholarly insights on the social lives of individuals with ADRD, there are four major limitations in the literature. First, small samples of individuals with dementia limit generalizability and understanding of causal links between ADRD diagnosis and outcomes. Second, the vast majority of previous studies compared the social outcomes of people with and without ADRD, preventing scholars from parsing out how receiving a diagnostic label, not the symptoms of ADRD, per se, affects social relationships among those with this condition. Third, many studies examine a single indicator of social relationships, even though ADRD diagnoses affect a wide range of social outcomes, preventing the direct comparison of the magnitudes of associations between ADRD diagnosis and multiple social outcomes.^24^ And fourth, previous studies did not focus on ethnoracial variations in the effects of a diagnostic label, despite evidence that non‐White people with dementia rely more on support from their informal social networks but engage in fewer social activities.^25^, ^26^, ^27^, ^28^, ^29^, ^30^ Because of historical racism and discrimination experienced by non‐White individuals, the practice of giving a diagnosis of ADRD may not lead to increases in support by professionals and decreases in social engagement and social networks among them. This may be exacerbated by ethnoracial disparities in knowledge of ADRD, leading to stronger misconceptions and stigmatization in non‐White groups.^31^ In fact, previous studies suggested disparities in the clinical consequences of a diagnosis of ADRD by showing that people of color are less likely to be prescribed with antidementia medicines,^32^, ^33^, ^34^ spend less out‐of‐pocket costs related to ADRD,^35^ and are more likely to have serious psychological distress due to a diagnosis of ADRD^36^ compared to their White counterparts. However, it is not clear if there exist heterogeneous effects of a diagnostic label of ADRD on socio‐behavioral constructs across different ethnoracial groups.

To address these gaps, this study aimed to examine the effects of a diagnostic label of dementia on a range of social relationships using a large nationally representative sample. Particular attention was paid to the impact of an ADRD diagnosis on a range of social relationships. It also aimed to test whether the effects of a diagnostic label of ADRD varied across ethnoracial groups.

**RESEARCH IN CONTEXT**

1. **Systematic review**: The authors reviewed the literature using traditional sources. Despite the scholarly insights on the social lives of individuals with ADRD, there are four major limitations in the literature. First is the use of small samples of people with a diagnosis of dementia. Second, the vast majority of previous studies did not examine how receiving a diagnostic label, not the symptoms of ADRD, per se, affects social relationships among those with this condition. Third, there is no study that assesses a comprehensive set of social outcomes. Finally, previous studies did not focus on ethnoracial variations in the effects of a diagnostic label.
2. **Interpretation**: A diagnostic label of ADRD may increase informal and formal social support, while it may decrease some aspects of social engagement. It also may decrease the likelihood of having a good friend in the neighborhood. The effects of a diagnostic label of ADRD on social relationships may be largely similar across ethnoracial groups, with some exceptions. Findings suggest that social and healthcare service professionals should identify necessary strategies to maintain or improve social support, engagement, and networks after the diagnosis of ADRD to improve individual outcomes.
3. **Future directions**: Future research that examines the mechanisms between a diagnostic label of ADRD and social relationships will help to identify specific strategies for maximizing positive impacts and minimizing negative ramifications of the diagnosis.

## 2 METHODS

### 2.1 Data and sample

Data were drawn from the Health and Retirement Study (HRS). The HRS is a nationally representative and longitudinal study that has been biennially surveying more than 20,000 adults aged 51 and older and their spouses since 1992. Core HRS interviews were conducted in participants’ homes or via telephone. We utilized the data collected between 2000 and 2018. We created a pooled sample with a pair of baseline and follow‐up waves (2000 and 2002, 2002 and 2004, …, and 2016 and 2018).

A total of 175,805 person‐year observations were included in the initial pooled sample. Five exclusion criteria were applied to obtain the final sample for this study. First, respondents who were younger than 70 years at baseline were excluded (*n* = 104,150). We used this age criterion because we utilized an algorithm to calculate the probability of having dementia status, which is only applicable to people 70 years or older.^37^ Second, we excluded participants who were predicted *not* to have dementia status at either baseline or follow‐up (*n* = 64,305). Dementia status was determined using Gianattasio‐Power predicted dementia probability scores and dementia classifications.^37^ We used the machine‐learning LASSO model, which utilizes 56 indicators to predict the probability of having dementia status among HRS participants who were 70 or older, including sociodemographic, cognition, physical functioning and health, health behavior, and social engagement variables. We utilized this algorithm because it was developed to improve the sensitivity, specificity, and overall accuracy of its predictive performance across three ethnoracial groups (non‐Hispanic White, non‐Hispanic Black, and Hispanic) in comparison to earlier algorithms (eg, Herzog‐Wallace,^38^ Langa‐Weir,^39^ and Hurd^21^ algorithms).^37^, ^40^ Its race/ethnicity‐specific cut‐off points are shown to perform comparably across ethnoracial groups, making it ideally suited for health disparities research.^37^ This algorithm is trained and evaluated using the subsample of the HRS called Aging, Demographics, and Memory Study (ADAMS), in which detailed clinical assessments of ADRD are available.^41^ The race/ethnicity‐specific cut‐off points of the LASSO model have good overall psychometric properties: sensitivity 83%, specificity 92%, and accuracy 91%. Third, we excluded participants who had ethnoracial identities other than non‐Hispanic White, non‐Hispanic Black, or Hispanic because the Gianattasio‐Power algorithm does not have a cut‐off point for them due to low sample sizes in the HRS. Fourth, we also excluded those who did not have back‐to‐back years of HRS participation (*n* = 81). And fifth, we excluded participants who had a diagnosis of dementia at baseline but did not report having it at follow‐up (*n* = 110). The final sample consisted of 7,159 person‐year observations (3,563 unique participants).

### 2.2 Measures

#### 2.2.1 Diagnosis of ADRD

The independent variable of the current study was having a diagnostic label of ADRD at follow‐up. HRS asks two separate questions about whether a doctor has ever told respondents that they have *“Alzheimer's disease”* or *“dementia, senility, or any other serious memory impairment.”* The response of “yes” to either question was recorded as having a diagnostic label of ADRD. The variable was dichotomously coded where those who did not have a diagnostic label were coded as “0” and those who received a diagnosis were coded as “1.”

#### 2.2.2 Social relationships

This study included eight dependent variables at follow‐up, all conceptualized as social relationships (social support, social engagement, and social networks). For social support, four variables were included: getting help with household chores, receiving informal support for activities of daily living (ADL) or instrumental activities of daily living (IADL), receiving informal support for finance, and receiving formal support for ADL or IADL. For getting help with household chores, the HRS asked, *“Do you get any help with work around the house or yard because of your health problems?”* (“Yes” and “No” answer options.) Receiving informal support for ADL or IADL was measured by whether the respondent was helped by an unpaid helper in the last month with ADL and IADL, with the same “Yes” and “No” answer options. Since support for ADL help and IADL help is very different from support for financial matters, receiving informal help for finance was separately measured by whether the respondent was helped by an unpaid helper in the last month with finance, with the same “Yes” and “No” answer options. Similarly, receiving formal help for ADL or IADL was measured by whether the respondent was helped by a paid helper in the last month with ADL and IADL, with the same “Yes” and “No” answer options. Formal support for finance was not included because an extremely low percentage (0.7%) of participants were financially supported by a paid helper.

To measure social engagement, two variables were utilized: formal volunteering and attending religious services. For volunteering, the HRS asked, “*Have you spent any time in the past 12 months doing volunteer work for religious, educational, health‐related, or other charitable organizations?*” (“Yes” and “No” answer options.) For attending religious services, we used the question “*About how often have you attended religious services during the past year?*” Respondents indicated the frequency of attending religious services using a 5‐point scale: 1 = not at all, 2 = one or more times a year, 3 = two or three times a month, 4 = once a week, and 5 = more than once a week.

We included two variables of social networks: children living nearby and having good friends nearby. For children living nearby, the HRS asks *“Do any of your children who do not live with you live within 10 miles of you?”* (“Yes” and “No” answer options.) For having good friends nearby, the HRS asks *“Do you have any good friends living in your neighborhood?”* (“Yes” and “No” answer options.)

#### 2.2.3 Covariates

A range of covariates at baseline (the first wave in the two‐wave pairs) was identified as correlates of the probability of having a diagnostic label of ADRD, which were based on a thorough review of the literature.^42^, ^43^, ^44^, ^45^, ^46^, ^47^, ^48^ Covariates included age, gender, education, race/ethnicity, household income (transformed using the inverse hyperbolic sine), marital status, use of proxy respondent, having psychiatric problems, self‐rated memory, number of doctor visits, having no health insurance, living in a nursing home, living alone, and cognitive function. Cognitive function was measured using scores on telephone interviews for cognitive status (TICS‐27^39^). TICS‐27 is a scale of cognitive function with validated cut‐off points to detect dementia. Scores on TICS‐27 range from 0 to 27, where higher scores indicate higher cognitive function. Because TICS‐27 was not administered to participants who utilized proxy respondents, we included the short form of the informant questionnaire on cognitive decline in the elderly (IQCODE^49^). Summary scores on IQCODE range from 1 to 5, where higher scores indicate more cognitive impairment. Additionally, we included the year at baseline in the analysis to capture potential effects from increased dementia awareness and diagnosis over time.^50^ The same set of covariates was included in the model estimating the effect of a diagnostic label on social relationships. In this outcome model, we added a covariate of change in cognitive function. Change in cognitive function was measured using the Langa‐Weir classification.^39^, ^51^ Based on this classification, we categorized people into “no dementia” and “dementia” at each wave. The changing variable was constructed as follows: 1 = no dementia at both waves, 2 = no dementia at baseline but had dementia at follow‐up, 3 = had dementia at both waves, 4 = had dementia at baseline but no dementia at follow‐up. Race/ethnicity was utilized to conduct subgroup analyses. We combined two questions, one for race and one for ethnicity, to create categories for Hispanic, non‐Hispanic White, and non‐Hispanic Black.

### 2.3 Statistical analysis

We utilized a two‐step quasi‐experimental process for this study. First, we created entropy balancing (EB) weights, a form of inverse probability of treatment (IPT) weighting using the WeightIt package in R, to generate weights that estimate the average treatment effect (ATE) of receiving a diagnostic label of ADRD. This method balances the “treatment” (having received a diagnostic label of ADRD) and “control” (not having received a diagnostic label of ADRD) groups on the means of the previously described pretreatment covariates^52^, ^53^ (ie, baseline covariates at baseline predict dementia diagnosis in the following wave). While a variety of covariate balancing methods exist, EB has been shown to be one of the most promising tools in its ability to better balance treatment and control groups in observational studies than earlier methods.^54^, ^55^, ^56^

As a form of sensitivity analysis, we also employed generalized boosted modeling (GBM) to generate IPT weights using the RAND TWANG machine learning program in R, a machine‐learning method that has been shown to have lower prediction error than traditional binary logistic regression methods.^57^ Here, we used the same pretreatment covariates as the EB model (except for TICS‐27 and IQCODE) and, after assessing several iterations, chose a model that allowed for up to three interactions between the covariates with a shrinkage of 0.001 and 100,000 trees (more information on TWANG can be found at the RAND website^58^).

**FIGURE 1:**
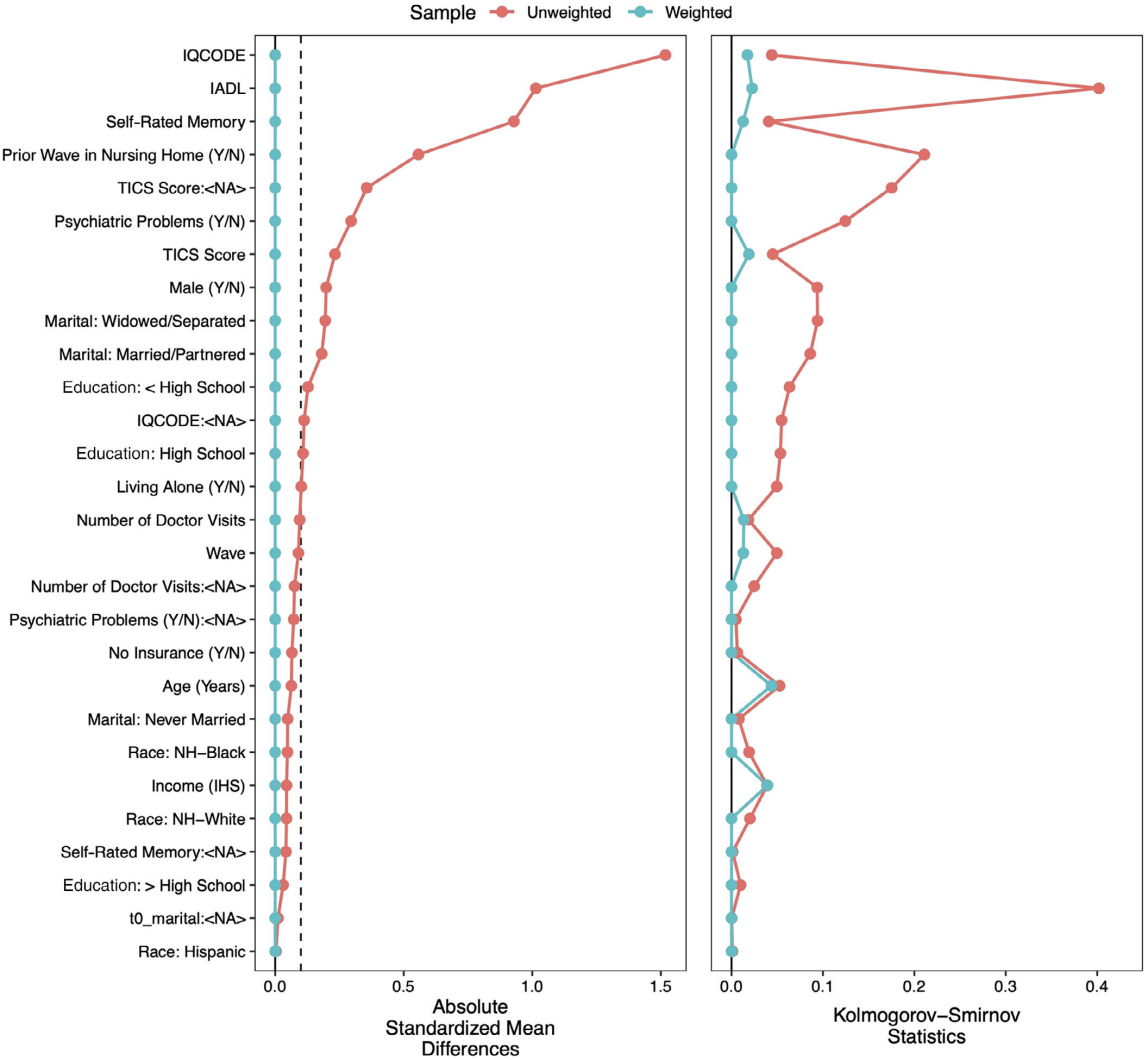
Entropy balancing‐weighted covariate balance between treatment and control groups.

To compare the weights from both the EB and GBM models, we considered the absolute standardized mean differences (ASMD) as well as the Kolmogorov‐Smirnov (KS) statistics between the covariates in the treatment and control groups.^59^ The ASMD, on a standardized scale so as to increase comparability between covariates, assesses the distance between the means of the treatment and control groups. The KS statistics provide a comparison of the distribution of the covariates between treatment groups. The results, shown in Figures 1 (EB) and 2 (GBM), indicate that the EB weights were superior in terms of their abilities to keep all ASMDs below the generally accepted threshold of 0.1^56^ while also better minimizing the KS statistics. (The figures were created using the cobalt package in R.^60^) We also conducted bivariate hypothesis tests to assess balance, as shown in Table 1. Here, too, the EB weights were superior, with not a single statistically significant difference between the treatment and control groups on the range of covariates. (We note that the GBM weighting still improved the balance on most measures, just not as well as EB weighting.) As a result of these tests, we decided to use the EB weights in our outcome analysis, resulting in an effective sample size of 2,248.72 for the treatment and 1,803.32 for the control groups.

**FIGURE 2:**
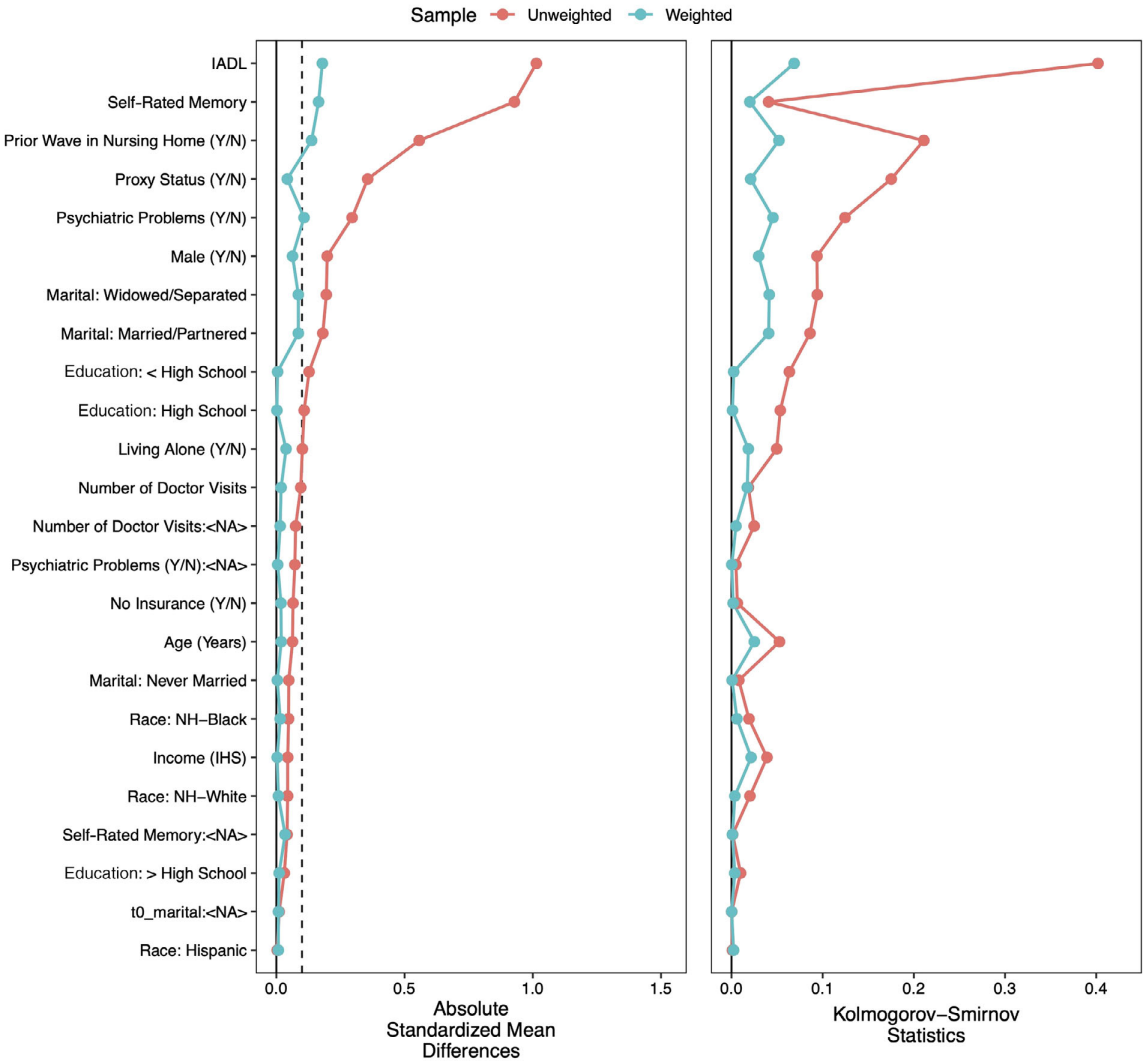
Generalized boosted modeling‐weighted covariate balance between treatment and control groups.

**TABLE 1:** Comparisons between treatment and control groups before and after weighting.

	Unweighted	EB Weighted	GBM Weighted
	*P*	*P*	*P*
Wave	<0.001*	0.999	0.015^a^, *
Age (Years)	0.008*	0.998	0.565
Male	<0.001*	1.000	0.042*
Race (*ref*: NH White)			
NH Black	0.039*	0.999	0.642
Hispanic	0.621	0.997	0.863
Education (*ref*: < High school)			
High school	<0.001*	0.999	0.969
> High school	0.004*	0.998	0.738
Income (IHS)	0.063	0.999	0.902
Marital status (*ref*: Married or partnered)			
Divorced or widowed	<0.001*	0.998	0.004*
Never married	0.786	1.000	0.587
IADL	<0.001*	0.997	<0.001*
Proxy status	<0.001*	0.997	0.192
Psychiatric problems	<0.001*	1.000	0.001*
Self‐rated memory	<0.001*	0.998	<0.001*
Number of doctor visits	0.001*	0.999	0.682
Having no insurance	0.008*	0.997	0.521
Nursing home previous wave	<0.001*	0.998	<0.001*
Living alone	<0.001*	1.000	0.236
TICS score (self only)	<0.001*	0.999	<0.001^a^, *
IQCODE (proxy only)	<0.001*	0.999	<0.001^a^, *

*Notes*: Sample created using the Gianattasio‐Power algorithm (*N* = 7,159). Balance assessed on a series of logistic regressions.

Abbreviations: EB, entropy balancing; GBM, generalized boosted modeling; HIS, inverse hyperbolic since; IADL, instrumental activities of daily living; IQCODE, informant questionnaire on cognitive decline in the elderly; NH, non‐Hispanic; TICS, telephone interview for cognitive status.

^a^ Wave, TICS score, and IQCODE were not included in the final GBM weighting equation because their inclusion worsened the absolute standardized mean differences for important variables, including TICS score and IADL.

* *p* < 0.05.

Then we employed an outcome‐wide analytic (OWA) approach to account for correlations among outcomes while yielding individual coefficients for each outcome. The weights generated from the IPT weighting process were included in this analysis, forming a “doubly robust” process of weighting *and* covariate control that has been shown to reduce bias in results.^61^, ^62^ In this OWA approach, multiple generalized estimating equations were used to regress each social relationship outcome on ADRD diagnosis. We estimated risk ratios (RRs) for the outcomes. For binary outcomes with more than 10% prevalence, a log link and Poisson distribution was used. For binary outcomes with 10% or lower prevalence (ie, volunteering), a log link with binary logistic distribution was employed.^63^ Ordinal logistic regression was used for an ordinal outcome (religious participation). Since multiple outcomes were tested in the same model, we used a Bonferroni correction to adjust for multiple testing. For all analyses, we adjusted for demographic variables, health behaviors, other health issues, and prior values of the outcome variables along with the IPT weighting. All missing data on the exposure, covariates, and outcomes were imputed using multiple imputation by chained equations (mice package in R), and 10 datasets were created. This approach provides more advantages compared to other strategies for handling missing data and addresses some issues due to attrition.^64^

Several supplementary analyses were conducted to test the robustness of the final analytic model. First, models were reanalyzed with different sets of covariates. Additional covariates include self‐reported health (fair or poor), alcohol consumption (three or more drinks daily), smoking (currently smoking), a number of chronic conditions (0 to 5), and physical activity (vigorous activity daily). They were not considered in the final model because the variables consistently yielded non‐significant effects, and including these variables did not change the substantive conclusions. Further, some variables were highly correlated with the existing covariates (eg, chronic conditions and IADL) or had a small sample size issue (eg, lack of physical activity among people with ADRD). In all cases, we found similar results as our final published findings. Second, complete case analyses were conducted to evaluate the effects of multiple imputations. Most missingness occurred for attending religious services, receiving formal support, and a number of doctor visits. Although the patterns were similar to the final analytic model, complete case analyses yielded non‐significant findings except for attending religious services and receiving formal support.

## 3 RESULTS

Thirty‐three (46.6%) respondents had a diagnostic label of ADRD at follow‐up. There was no statistically significant difference in the proportion of having a diagnostic label of ADRD across ethnoracial groups. Table 2 illustrates the sample characteristics and ethnoracial differences at baseline. All the variables of social relationships except children living nearby were significantly different across ethnoracial groups at baseline. The non‐Hispanic Black group had the lowest percentage of people who get help with household chores, while this group had the highest percentages of people who attended religious services more than once a week and those who had friends nearby. The non‐Hispanic White group had the highest percentage of people who spent time volunteering. The Hispanic group had the highest percentage of people who had one or more informal or paid helpers for ADL/IADL and financial support. Among the covariates, proxy‐assessed cognitive function (IQCODE) and the number of doctor visits were the only variables that were not statistically significantly different across ethnoracial groups.

**TABLE 2:** Sample characteristics and baseline comparison across ethnoracial groups.

	All	White	Black	Hispanic	
	M/%	M/%	M/%	M/%	*P*
Diagnostic label of dementia					
No diagnosis	53.4	52.7	55.8	53.8	0.099
Have diagnosis	46.6	47.3	44.2	46.3	
Get help					
No	52.2	53.4	48.2	52.8	0.005
Yes	47.8	46.6	51.8	47.2	
Informal support for ADL/IADL					
No helper	57.0	60.3	52.9	42.6	<0.001
One or more helpers	43.0	39.7	47.1	57.4	
Informal support for finance					
No helper	62.5	62.9	63.2	58.1	0.035
One or more helpers	37.5	37.1	36.8	41.9	
Formal support for ADL/IADL					
No paid helper	86.7	88.4	85.9	76.4	<0.001
One or more paid helpers	13.3	11.6	14.1	23.6	
Volunteering					
No volunteering	90.8	89.8	91.4	96.6	<0.001
Volunteered	9.2	10.2	8.6	3.4	
Attend religious services					
Not at all	33.9	35.5	25.2	40.5	<0.001
One or more times a year	17.8	17.6	19.2	16.8	
Two or three times a month	11.6	10.5	15.9	9.7	
Once a week	26.7	27.4	26.2	22.7	
More than once a week	10.1	9.0	13.5	10.3	
Children live nearby					
No	40.7	41.0	41.1	37.4	0.171
Yes	59.3	59.0	58.9	62.6	
Have friends nearby					
No	40.8	40.5	36.9	50.7	<0.001
Yes	59.2	59.5	63.1	49.3	
Age	83.2	83.6	82.2	82.9	<0.001
Gender					
Female	65.3	64.0	69.2	66.6	0.001
Male	34.7	36.0	30.9	33.4	
Education					
Less than high school	46.1	35.6	63.3	83.2	<0.001
High School	42.3	49.4	31.3	15.4	
More than high school	11.6	15.0	5.5	1.4	
Household income					
Lowest	25.0	17.2	42.3	43.3	<0.001
Second lowest	25.0	22.7	28.5	33.6	
Second highest	25.0	28.4	17.4	17.3	
Highest	25.0	31.7	11.8	5.9	
Marital status					
Married	35.4	38.6	23.1	38.2	<0.001
Widowed separated	61.8	59.5	71.7	57.3	
Never married	2.8	1.9	5.3	4.5	
Number of IADL difficulty	1.9	1.9	2.0	2.3	<0.001
Cognitive function (TICS‐27)	6.3	6.9	5.2	4.8	<0.001
Cognitive function (IQCODE)	3.6	3.7	3.6	3.7	0.930
Proxy					
Not proxy	52.4	52.1	57.4	44.6	<0.001
Proxy respondent	47.6	47.9	42.6	55.4	
Psychiatric problem					
Do not have a problem	76.6	75.4	81.9	74.2	<0.001
Have psychiatric problems	23.4	24.6	18.1	25.8	
Self‐rated memory	2.4	2.4	2.4	2.2	<0.001
Number of doctor visits	13.3	13.1	13.8	13.5	0.723
Insurance					
Have insurance	99.0	99.3	98.6	98.0	0.001
No insurance	1.0	0.7	1.4	2.1	
Nursing home					
Not living in nursing home	81.6	79.1	85.9	89.6	<0.001
Living in nursing home	18.5	20.9	14.1	10.4	
Living arrangement					
Living with someone	60.7	57.7	63.6	75.3	<0.001
Living alone	39.3	42.3	36.4	24.7	

*Notes*: Values for age, household income, IADLs, and doctor visits are means; values for all other variables are percentages.

Abbreviations: ADL, activities of daily living; IADL, instrumental activities of daily living; IQCODE, informant questionnaire on cognitive decline in the elderly; TICS, telephone interview for cognitive status.

Table 3 summarizes the results of the propensity‐score‐weighted outcome‐wide analysis of the effects of a diagnostic label of ADRD on eight variables of social relationships. Having a diagnostic label of ADRD was significantly associated with higher likelihood of getting help with household chores (RR [95% confidence interval (CI)] = 1.157 [1.131, 1.184], *p* < 0.001), receiving informal support for ADL or IADL (RR [95% CI] = 1.349 [1.320, 1.379], *p* < 0.001), receiving informal support for finance (RR [95% CI] = 1.849 [1.804, 1.896], *p* < 0.001), and receiving formal support for ADL or IADL (RR [95% CI] = 1.505 [1.427, 1.588], *p* < 0.001) at follow‐up. In contrast, having a diagnostic label of ADRD was significantly associated with lower social engagement such as volunteering (OR [95% CI] = 0.458 [0.389, 0.539], *p* < 0.001) and attending religious services (OR [95%CI] = 0.892 [0.870, 0.913], *p* < 0.001) at follow‐up. Having a diagnostic label of ADRD was significantly associated with a lower likelihood of having a good friend nearby (RR [95% CI] = 0.805 [0.782, 0.829], *p* < 0.001), while it did not have a significant effect on the likelihood of having children nearby (RR [95% CI] = 1.011 [0.992, 1.031], *p* = 0.261).

**TABLE 3:** Results of outcome‐wide analysis of effects of diagnostic label on social relationships and subgroup analyses.

		95% CI				
	Class	Est.	Lower	Upper	*P*	Sig.
Entire sample (*n* = 7,152)						
Get help	Common Binary	1.157	1.131	1.184	<0.001	***
Informal support for ADL/IADL	Common Binary	1.349	1.320	1.379	<0.001	***
Informal support for finance	Common Binary	1.849	1.804	1.896	<0.001	***
Formal support for ADL/IADL	Common Binary	1.505	1.427	1.588	<0.001	***
Volunteering	Rare Binary	0.467	0.408	0.536	<0.001	***
Attend religious services	Ordinal	0.896	0.879	0.914	<0.001	***
Children live nearby	Common Binary	1.003	0.986	1.019	0.763	N.S.
Have friends nearby	Common Binary	0.830	0.811	0.850	<0.001	***
White respondents (*n* = 4,994)						
Get help	Common Binary	1.125	1.093	1.158	<0.001	***
Informal support for ADL/IADL	Common Binary	1.404	1.364	1.446	<0.001	***
Informal support for finance	Common Binary	1.948	1.891	2.007	<0.001	***
Formal support for ADL/IADL	Common Binary	1.678	1.563	1.802	<0.001	***
Volunteering	Rare Binary	0.458	0.389	0.539	<0.001	***
Attend religious services	Ordinal	0.892	0.870	0.913	<0.001	***
Children live nearby	Common Binary	1.011	0.992	1.031	0.261	N.S.
Have friends nearby	Common Binary	0.805	0.782	0.829	<0.001	***
Black respondents (*n* = 1,465)						
Get help	Common Binary	1.125	1.073	1.178	<0.001	***
Informal support for ADL/IADL	Common Binary	1.320	1.264	1.378	<0.001	***
Informal support for finance	Common Binary	1.771	1.672	1.875	<0.001	***
Formal support for ADL/IADL	Common Binary	1.397	1.237	1.579	<0.001	***
Volunteering	Rare Binary	0.550	0.413	0.733	<0.001	***
Attend religious services	Ordinal	0.908	0.873	0.944	<0.001	***
Children live nearby	Common Binary	0.989	0.952	1.028	0.576	N.S.
Have friends nearby	Common Binary	0.842	0.805	0.880	<0.001	***
Hispanic respondents (*n* = 733)						
Get help	Common Binary	1.080	1.007	1.159	0.032	*
Informal support for ADL/IADL	Common Binary	0.375	0.166	0.847	0.018	*
Informal support for finance	Common Binary	0.810	0.755	0.870	<0.001	***
Formal support for ADL/IADL	Common Binary	0.982	0.932	1.034	0.488	N.S.
Volunteering	Rare Binary	1.024	0.947	1.107	0.557	N.S.
Attend religious services	Ordinal	1.247	1.189	1.308	<0.001	***
Children live nearby	Common Binary	1.612	1.502	1.730	<0.001	***
Have friends nearby	Common Binary	1.232	1.100	1.379	<0.001	***

Abbreviations: ADL, activities of daily living; Est., Estimate, risk ratios for common and rare binary outcomes and odds ratios for ordinal outcome; IADL, instrumental activities of daily living; IQCODE, informant questionnaire on cognitive decline in the elderly; N.S., not significant; Sig., significance; TICS, telephone interview for cognitive status.

* *p* < 0.05 before Bonferroni correction; **p < 0.01 before Bonferroni correction; ***p < 0.05 after Bonferroni correction (the *p* value cut‐off for Bonferroni correction is *p* = 0.05/8 outcomes = *p* < 0.006. All common binary outcomes (prevalence > 10%) were estimated via modified Poisson regression with robust standard error. Rare binary outcome (prevalence ≤10%) were estimated using binary logistic regression with robust standard error.

Table 3 also shows the results of subgroup analyses. We found similar findings in the analysis with the non‐Hispanic White‐only and non‐Hispanic Black‐only samples. On the other hand, for Hispanic individuals the effects of a diagnostic label on getting help with household chores (RR [95% CI] = 1.080 [1.007, 1.159], *p* = 0.032), receiving informal support for ADL or IADL (RR [95% CI] = 0.375 [1.007, 1.159], *p* = 0.018), receiving formal support for ADL or IADL (RR [95% CI] = 0.982 [1.007, 1.159], *p* = 0.488), and volunteering (RR [95% CI] = 0.432 [0.231, 0.810], *p* = 0.009) were not significant. Contrary to the results with other groups, for Hispanic individuals a diagnostic label was associated with decreased likelihood of receiving informal support for finance (RR [95% CI] = 1.080 [1.007, 1.159], *p* = 0.032) and increased likelihood of attending religious services (RR [95% CI] = 0.810 [0.755, 0.870], *p* = < 0.001), having children live nearby (RR [95% CI] = 1.612 [1.502, 1.730], *p* = < 0.001), and having friends nearby (RR [95% CI] = 1.232 [1.100, 1.379], *p* = < 0.001).

## 4 DISCUSSION

As a highly stigmatized condition, receiving a diagnostic label of ADRD may profoundly affect the social lives of recipients. However, the effects of a diagnostic label of ADRD on social relationships have not been well investigated. Our study moves the field forward in five key ways. First, our sample only included people predicted to have ADRD through the use of Gianattasio‐Power‐predicted dementia probability scores and dementia classifications shown to be 91% accurate.^37^ Second, we included a large sample of people predicted to have ADRD by pooling multiple years of data together, increasing statistical power. Third, we created a quasi‐experimental study by balancing those who had received a diagnostic label of ADRD with those who had not on multiple factors that have been shown to be related to a diagnosis^42^, ^43^, ^44^, ^45^, ^46^, ^47^, ^48^ through EB. Fourth, we considered a broader range of potential social relationship outcomes using an OWA approach to model multiple dependent variables at once to account for correlations among them.^24^ And fifth, we considered ethnoracial variations in the results to account for disparities in access to care and outcomes by race and ethnicity among people with ADRD.^32^, ^33^, ^34^, ^35^, ^36^

A strength of our study is its use of EB, which balanced those with and without a diagnosis of ADRD on multiple factors, offering strong evidence for the impact of an ADRD diagnosis on social bonds. Caution should be exercised, however, in connection with the limitations of EB and similar methods, as they do not adjust for unobservable factors. Further, the comparison across three ethnoracial groups at baseline demonstrates the benefits of utilizing the Gianattasio‐Power algorithm with a diverse ethnoracial sample. Non‐Hispanic Black and Hispanic individuals had higher paid helper percentages for ADL or IADL than Whites, in contrast to prior findings, possibly due to more severe dementia symptoms. In fact, average numbers of IADL difficulty were slightly higher and cognitive function was lower among non‐Hispanic Black and Hispanic individuals than non‐Hispanic White individuals. Furthermore, there were no significant differences between ethnoracial groups regarding ADRD labels, which is intriguing given prior studies indicating lower likelihood of having ADRD diagnoses in cognitively impaired non‐Hispanic Black and Hispanic individuals.^46^, ^50^ These observations could underscore the precision of the Gianattasio‐Power algorithm and its demonstrated strength in non‐White samples.^40^

Regarding the relationship between a diagnostic label of ADRD and various aspects of social relationships, our findings suggest that a diagnostic label of ADRD may increase the receipt of social support, while decreasing the likelihood of engaging in social activities such as volunteering and attending religious services. Results also indicate the general lack of ethnoracial heterogeneity in the relationship between a diagnostic label of ADRD and social relationship profiles.

The obtained results suggest that a diagnostic label of ADRD may increase the likelihood of getting help with household chores and receiving informal and formal support on healthcare service utilization.^18^, ^19^, ^21^, ^65^ We advance the evidence by showing, with a quasi‐experimental study design, that a diagnostic label of ADRD may contribute to increasing informal and formal social support among people with ADRD. Interestingly, this is partially inconsistent with a previous study that revealed that dementia diagnoses did not change perceived social support.^22^ Although a direct comparison may not be possible, this inconsistency implies that unmet needs may still remain despite increases in support after a diagnosis.^66^, ^67^ Given that unmet needs of people with ADRD are more likely to be social than medical or clinical,^66^, ^67^, ^68^, ^69^ social support increased by a diagnostic label may be limited to certain aspects of support.

Our findings also indicated that a diagnostic label of ADRD may reduce the social engagement of recipients. This is consistent with relatively rich evidence of social disengagement among people with ADRD.^22^, ^23^, ^70^, ^71^, ^72^ This study strengthened the evidence by utilizing a larger sample and more robust statistical methods. It is also consistent with the emerging trend of research that challenges the traditional “deficit model” of dementia, which claims that self‐withdrawal due to impairment caused by ADRD accounts for social disengagement among people with this condition.^68^, ^73^ Our findings and previous studies suggest a diagnostic label (ie, stigma) as a major driver of social disengagement among people with ADRD rather than impairment.

We found no significant effect of a diagnostic label of ADRD on the likelihood of children living nearby. One possible reason is that relocation of either children or the person with a diagnostic label is often a slow process and requires more time than changes in social support and social engagement. The null effect of a diagnostic label may be due to the relatively short (2 years) follow‐up time in this study. On the other hand, we found that the diagnostic label of ADRD reduced the likelihood of having a good friend in the neighborhood. This may be due to the stigma of friends feeling uncomfortable visiting the recipient of a diagnostic label of ADRD or the recipient feeling fear of communicating with friends.^68^, ^70^

Subgroup analyses suggest consistent ADRD effects on social bonds for non‐Hispanic White and non‐Hispanic Black groups, but less so for Hispanics. This has two implications. First, early diagnosis is crucial for effective support. As shown in our findings, once diagnosed, non‐Hispanic White and non‐Hispanic Black are both likely to receive more informal and formal social support. Nevertheless, ADRD is often undetected and underdiagnosed,^46^, ^47^ especially in ethnoracial minorities.^74^, ^75^ Strategies for timely detection across ethnoracial groups are vital. Second, more support for ethnoracial minorities may be needed to alleviate the negative impacts of a diagnosis of ADRD on social engagement and social networks. Studies have shown that non‐Hispanic Black older adults are more likely to be socially disengaged and isolated than their non‐Hispanic White counterparts.^25^, ^26^, ^27^, ^28^, ^29^, ^30^ If a diagnostic label of ADRD similarly reduces social engagement and social networks, non‐Hispanic Black individuals may be more likely to have negative consequences by such a label.

We found contradictory results with the Hispanic‐only sample. For Hispanic individuals, a diagnosis of ADRD may not increase the likelihood of receiving formal support for ADL or IADL, while it may increase social engagement and social networks. However, the findings with the Hispanic sample do not necessarily indicate the actual existence or lack of effects; rather, it might be that the current study did not contain a large enough sample of Hispanic individuals. Future studies should provide a more comprehensive understanding of the ethnoracial variations in the effects of a diagnostic label on social and healthcare service utilization.

This study has some limitations. First, although we utilized a nationally representative sample, the sample size may still not be large enough for ethnoracial subgroup analysis. The Hispanic sample (*n* = 733) may have suffered from insufficient statistical power considering that OWA estimates many parameters. Second, we did not include a formal diagnosis of ADRD as an independent variable. We were unable to differentiate between having a formal diagnosis and knowing about it. Using these, we could separate the effect into a clinical diagnosis and disclosure of it. Future studies are needed to examine the impact of a diagnosis of ADRD using formal documentation of the diagnosis. Third, how the diagnosis was disclosed was not measured in this study, even though there are techniques to minimize psychological distress caused by a diagnosis.^7^ Future studies should examine to what extent the methods of disclosure influence the effect of a diagnostic label on social outcomes. Fourth, we did not capture the duration of ADRD diagnosis. Given that HRS collects data biennially, it lacks information regarding the precise timing of participants being informed about their ADRD diagnosis within the 2‐year intervals. This gap could impact the results, particularly considering that certain outcome variables pertain to behaviors observed over the preceding 12 months. While our findings are rooted in the most reliable data and analysis available, future research must gather more precise information on the timing of ADRD diagnoses to corroborate the conclusions from the current study. Finally, it should be noted that EB does not account for unobservable characteristics. There may be a significant confounding factor that was not measured in HRS. Nevertheless, we believe that the quasi‐experimental method we employed is the best available for causal inference between a diagnostic label and social relationships, considering that it is not possible to experimentally investigate the relationship.

The findings of this study are valuable. A diagnostic label of ADRD may increase social support, while it may decrease some aspects of social engagement. It also may decrease the likelihood of reporting a good friend in the neighborhood. The effects of a diagnostic label of ADRD may be largely similar across ethnoracial groups, with some exceptions. Findings suggest that social and healthcare service professionals should identify necessary strategies to maintain or improve social support, engagement, and networks after the diagnosis of ADRD to improve individual outcomes. Future research that examines the mechanisms between a diagnostic label of ADRD and social relationships will help to identify specific strategies for maximizing positive impacts and minimizing negative ramifications of the diagnosis.

## CONFLICT OF INTEREST STATEMENT

The authors declare no conflicts of interest. Author disclosures are available in the supporting information.

## CONSENT STATEMENT

Study subjects and their informants provided written informed consent to participate in the HRS. The study protocols of HRS were approved by the University of Michigan Institutional Review Board.

## Supporting information

supporting information
